# COVID-19 numbers and models: misleading us, or leading us out of misery?

**Published:** 2020-09-01

**Authors:** Heiko Philippin, Karin M Knoll, David Macleod

**Affiliations:** 1 Clinical Research Fellow: International Centre for Eye Health, London School of Hygiene & Tropical Medicine, UK, Global Advisor for Inclusive Eye Health/Research & Training: CBM, Bensheim, Germany, and Glaucoma Specialist: Eye Center, Medical Center, Faculty of Medicine, University of Freiburg, Germany.; 2 Consultant Ophthalmologist: Kilimanjaro Christian Medical Centre, Moshi, Tanzania and CBM, Bensheim, Germany.; 3 Assistant Professor: MRC Tropical Epidemiology Group, London School of Hygiene & Tropical Medicine, UK.

**The majority of the world’s population is affected to some degree or another by the COVID-19 pandemic and measures to curb its spread. Newspapers and social media bombard us with numbers, graphs, and predictions every day, but what do these numbers really tell us?**

**Figure:**
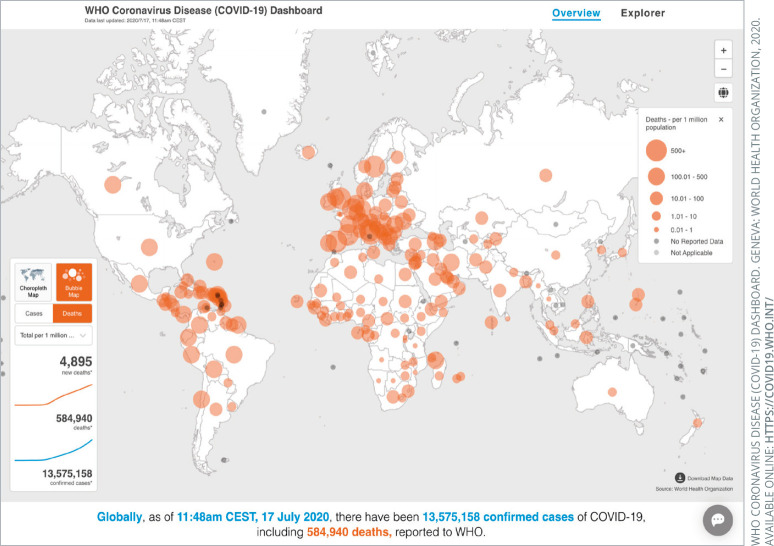
The World Health Organization map shows how countries are affected by COVID-19.

COVID-19 numbers and statistics have been dominating our news since the beginning of 2020. Although they fail to tell the story of the individual hardship and grief that many are dealing with, they can provide a bigger picture of the situation and assist in planning.

## How many people worldwide have been infected with COVID-19?

A common number which is reported is the count of positive test results for SARS-CoV-2. The number is collected by hospitals, laboratories or individual doctors and sent to national health agencies. A global overview is provided by the World Health Organization (**https://covid19.who.int/**) and other data portals. Newspapers publish daily updates and often countries are compared, or even ranked, by this number.

**“If little testing is being done, a country may have few positive test results but might still have many infected individuals.”**

However, this number of positive test results is not the same as the actual number of people infected with SARS-CoV-2. An unknown number of undetected infections exists in every country as:

- Some people who are infected do not have symptoms and are not tested
- Some people with symptoms do not have the opportunity to be tested. This could be due to a lack of testing kits or rules about who should be tested (e.g., different age and risk groups), and when they should be tested (e.g., immediately when showing symptoms or when hospitalised for COVID-19).

If little testing is being done, a country may have few positive test results but might still have many infected individuals.

It is also misleading to compare countries by the total number of people testing positive, because there are differences in **population size**.

**Example:** at a certain point during the pandemic, Honduras and Ireland both reported a total of 25,000 people with positive test results. However, when the number of tests in each country was compared with its population size, there were around **500** positive tests per 100,000 people in Ireland (population of about 5 million), compared with just **250** positive tests per 100,000 people in Honduras (population 10 million).

## How many people have died from COVID-19?

International comparisons of the number of COVID-19 deaths can be just as misleading, as countries use different definitions and policies regarding which deceased persons are counted. Some countries include only people who died of COVID-19 in hospital and tested positive for SARS-CoV-2, whereas others also include people who died outside the hospital or were suspected of having COVID-19. These definitions and policies may change over time within a country.

The so-called **excess mortality** can be helpful in finding out about the true impact of COVID-19 on death. Excess mortality looks at the number of *additional* deaths during a certain time period compared to the average number of deaths in the same period in previous years. Additional deaths may be directly attributed to COVID-19, but also indirectly if people with other conditions (such as heart attacks or strokes) did not receive the treatment they would normally have received. Excess mortality may be more meaningful than looking at the number of deaths reported to be directly due to COVID-19, as it does not depend on how COVID-19 deaths are recorded.

As we have seen, the number of positive test results or official COVID-19 deaths do not provide an accurate picture of the spread of the virus in a country. However, if the context and limitations are considered, they still provide a useful overview of the situation.

## What can models tell us?

Mathematical models aim to help us predict the effect of preventive measures and what may happen as an epidemic develops. These models can be informed by existing knowledge about the disease and the virus responsible, assumptions about how people are likely to behave, and data about the current situation, e.g., the number of people who are infected, have recovered, or have died.

The accuracy of these predictions will improve as the quality of the available data improves, and as we learn more about the virus, the disease it causes, and how people behave.

Mathematical modelling is useful because it can rapidly estimate the effects of different interventions, e.g., what might happen in a pandemic if the transmission of a virus is reduced by social distancing.^1^ However, for emerging diseases such as COVID-19, very little data exist initially, and modellers have to rely on untested assumptions. Scientists therefore model many different scenarios, based on different sets of assumptions, which also accounts for the wide range of possible outcomes we read about.

**“Overall, the number of people with a positive test result, and the number who have died, show only a part of the whole picture of the virus spread in a country.”**

## How do infectious diseases spread?

The spread of any infectious disease can be illustrated using established mathematical models. In the early phase of an epidemic or pandemic, growth is exponential. This means that it spreads slowly at first, but increases very rapidly later on.

**Example:** If one person with COVID-19 could infect three people a week, then there could be 3 new infections after one week, 9 infections after two weeks, 27 infections after three weeks, and 81 after four weeks. Six weeks later (after 10 weeks), there could be 59,049 infections!

As the virus spreads, more and more people become infected, recover, and – hopefully – develop some level of immunity. This reduces the number of people who are susceptible to infection. If there are low numbers of susceptible people in the population, then the chances of an infected individual coming into contact with susceptible individuals is reduced, in turn reducing the chances of onward transmission. This is known as herd immunity, an effect which has been observed less than anticipated during the SARS-CoV-2 pandemic so far. Using these categories of susceptible, infectious and recovered (or removed) people, a simplified model of a pandemic can be calculated (see panel). More testing for SARS-CoV-2 infection will help to provide better predictions of current and future scenarios.

**Understanding the dynamics of an epidemic**

**Figure 1:**
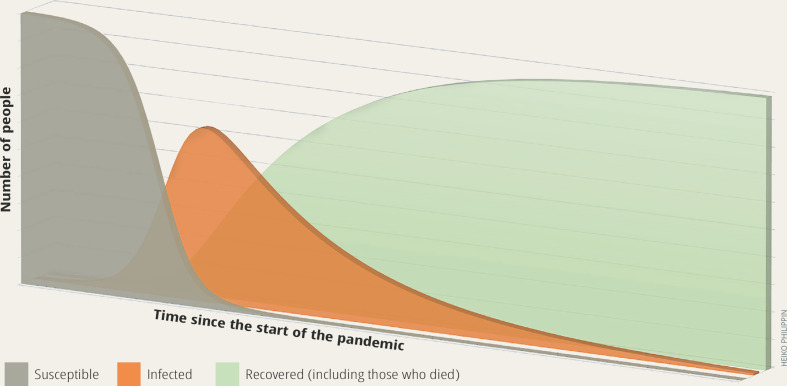
Susceptible-Infected-Recovered (SIR) model, showing how the pandemic develops over time

The Susceptible-Infectious-Recovered (SIR) model (Figure 1) shows how the SARS-CoV-2 virus spreads through a community or group of people.

During an outbreak, people can be grouped into three categories. Everybody starts in the group of persons who are susceptible and can be infected by the virus (grey shaded area). With time, more and more people become infected (orange shaded area). Protective measures such as social distancing or hand washing can slow down the spread. The third category (green) are people who have either recovered from the infection, or died.

Some of the parameters used in models have to be estimated; so predictions from models can therefore only be made with limited confidence. For example, the number of people who developed immunity against COVID-19 grew slower than anticipated in many regions.

## Containing viral spread and breaking chains of transmission

**Figure 2:**
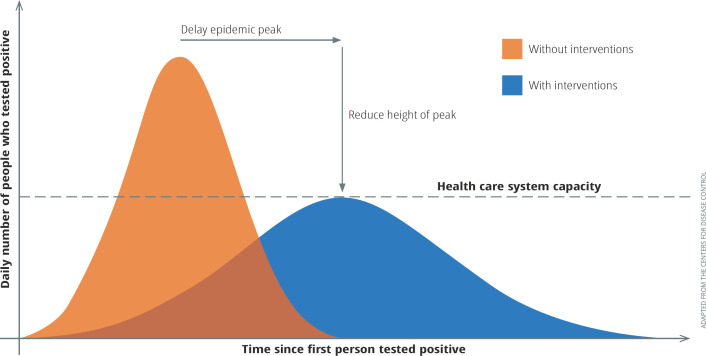
Modelling the impact of protective measures against the spread of COVID-19. Delaying and reducing the height of the peak (blue) ensures that the health system capacity is not exceeded.

In the context of the COVID-19 pandemic, the number of beds and health care personnel available to care for COVID-19 patients in intensive care units is known as the **health system capacity**, represented by the dashed horizontal line in Figure 2. The health system may become overwhelmed if the number of patients exceeds this, which increases the risk of patients dying from the disease. In order to manage the situation, the rate at which the virus spreads can be reduced by implementing protective measures that **reduce the risk of transmission** (e.g., thorough hand washing with soap and water and wearing a face covering in public) or **interrupt transmission** (e.g., by putting in place social isolation measures such as quarantine, or lock-down, in addition to other measures).

Slowing down the spread of the virus with these protective measures reduces the number of people infected at any one time, which changes the infection curve. Compare the curves in Figure 2. The blue curve (with interventions) is noticeably flatter and wider than the orange curve (without interventions). This is why it is sometimes referred to as “flattening the curve” of cases.

Even with a flattened curve, anyone who has not yet had COVID-19 will only be protected against the virus for as long as the protective measures or interventions continue. When measures are lifted, the virus can spread once again; this will only change once effective and affordable vaccines become available to most members of the community.

## Supporting complex decisions

Though of utmost importance, trying to reduce the number of patients who are severely ill at any one time is just one aspect in the management of the pandemic. It is intertwined with other factors which are important for the population as a whole. For example, a total lockdown can also have a detrimental effect on mental health or the ability of people to financially sustain themselves.

It is difficult to decide what level of preventive measures to take, and to maintain a balance between these and the overall functioning of society.^2^,^3^,^4^ A balanced and gradual exit strategy setting out when and how to reduce protective measures is necessary to avoid a new increase of infections (i.e., a second ‘wave’ or ‘peak’ in the blue curve, instead of the gradual reduction in infections currently shown in Figure 2); e.g., when preventive measures are not sufficient to prevent transmission, or if the virus is re-introduced from outside the community. Such decisions can be informed by data from test results and other sources as well as models based on these data.

In summary, a reliable set of data and mathematical models can help substantially to guide some of the complex decisions in this COVID-19 pandemic and – ultimately – to ease individual hardship.
